# ATF4 contributes to autophagy and survival in sunitinib treated brain tumor initiating cells (BTICs)

**DOI:** 10.18632/oncotarget.26569

**Published:** 2019-01-08

**Authors:** Sylvia Moeckel, Kelly LaFrance, Julia Wetsch, Corinna Seliger, Markus J. Riemenschneider, Martin Proescholdt, Peter Hau, Arabel Vollmann-Zwerenz

**Affiliations:** ^1^ Wilhelm Sander-NeuroOncology Unit and Department of Neurology, Regensburg University Hospital, Regensburg, Germany; ^2^ Department of Neuropathology, Regensburg University Hospital, Regensburg, Germany; ^3^ Department of Neurosurgery, Regensburg University Hospital, Regensburg, Germany

**Keywords:** glioblastoma, brain tumor initiating cells, ATF4, therapy resistance, receptor-tyrosine-kinase-inhibitor

## Abstract

Receptor tyrosine kinase (RTK) pathways are known to play an important role in tumor cell proliferation of glioblastoma (GBM). Cellular determinants of RTK-inhibitor sensitivity are important to optimize and tailor treatment strategies.

The stress response gene activating transcription factor 4 (ATF4) is involved in homeostasis and cellular protection. However, little is known about its function in GBM. We found that the ATF4/p-eIF2α pathway is activated in response to Sunitinib in primary tumor initiating progenitor cell cultures (BTICs). Furthermore, lysosome entrapment of RTK-inhibitors (RTK-Is) leads to accumulation of autophagosomes. In case of Sunitinib treated cells, autophagy is additionally increased by ATF4 mediated upregulation of autophagy genes. Inhibition of ATF4 by small interfering RNA (siRNA) reduced autophagy and cell proliferation after Sunitinib treatment in a subset of BTIC cultures.

Overall, this study suggests a pro-survival role of the ATF4/p-eIF2α pathway in a cell type and treatment specific manner.

## INTRODUCTION

Glioblastomas (GBMs) are the most common malignant brain tumors in the adult population, with a median survival time of about 15 months despite maximal therapy [1]. So far, Temozolomide, a cytotoxic drug in combination with radiation [2] and Optune, so-called Tumor Treating Fields [3], remain the only treatments that have improved outcome. Therefore, improved therapy options for patients with GBM are urgently needed.

With the increasing knowledge on tumor formation and progression in high grade gliomas, great expectations were raised in agents that target key oncogenic pathways, such as receptor tyrosine kinases (RTKs). With alterations in over 80% of GBMs, the RTK/PI3K/AKT pathway constitutes one of the most frequently altered groups of genes in this tumor type [4]. However, RTK inhibitors (RTK-Is) have shown negligible success in clinical trials against GBMs. No association between alterations within signaling pathways and the response to those drugs could be found [5, 6].

At the molecular level, mechanisms of acquired resistance have been described, among which are secondary mutations and activation of compensatory pro-survival signaling pathways (reviewed in [7, 8]).

During tumor progression, tumor cells encounter various environmental stresses like hypoxia and nutrient deprivation. In response to these stress conditions, cells activate a number of homeostatic pathways that are collectively known as the integrated stress response (ISR) [9].

ATF4 is the central transcriptional activator of the ISR, a program of gene expression involving multiple effectors that ultimately determine cell fate, depending on the severity and duration of stress as well as other micro-environmental factors [10]. The ISR pathway is initiated upon phosphorylation of the alpha subunit of eukaryotic initiation factor 2 (eIF2α) at serine 51. Four mammalian eIF2 kinases that phosphorylate eIF2α are known: general control non-derepressible 2 (GCN2), protein kinase R (PKR), heme-regulated eIF2α kinase (HRI) and PKR-like endoplasmic reticulum (ER) kinase (PERK), which is upregulated by ER stress [11].

Phosphorylation of eIF2α is accompanied by a global reduction of protein synthesis. Paradoxically, this event simultaneously leads to enhanced expression of ATF4, primarily via enhanced translation of its mRNA by a mechanism involving its 5′-UTR [12]. Previous studies have shown that ATF4 was significantly higher in all malignant tissues compared to the corresponding normal tissues [13].

It has been shown that genes involved in oxidative stress and nutrient uptake, but also components of the autophagic machinery are subject to ISR regulation [13–16]. Autophagosome formation and maturation is a highly regulated process that occurs through a series of distinct steps controlled by autophagy related genes (ATGs). Recent studies have shown that autophagy can allow cells to cope with stressors by destroying damaged proteins and organelles as a survival-promoting mechanism [17–19]. Autophagy activation has been observed during RTK-I treatment in various cancer cells [20].

The upregulation of ATF4 upon Sunitinib treatment was observed in a previously published genomic wide expression analysis of Sunitinib treated BTICs [21]. In the study presented here, we investigated the function of ATF4 in the context of treatment with the multi-targeted RTK inhibitor Sunitinib. We demonstrate that Sunitinib treatment activates the eIF2α/ATF4 and the autophagy pathway in GBM derived tumor cells and provide evidence of a causal link between both molecular events that constitute a ‘pro-survival’ function in a cell-type specific manner.

## RESULTS

### ATF4 expression is increased in response to sunitinib

**Figure 1:**
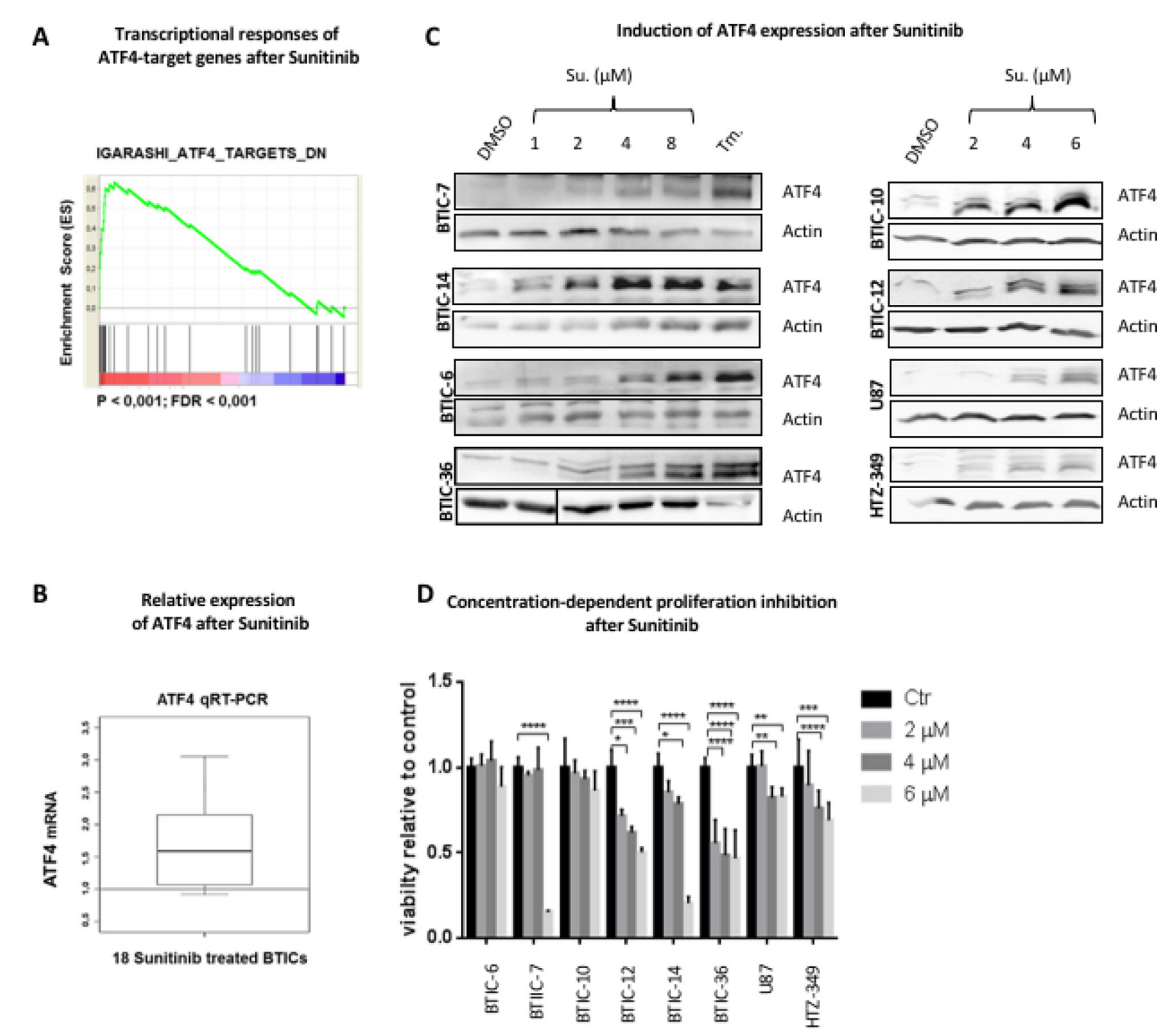
Expression of ATF4 after sunitinib treatment (**A**) Genome-scale gene expression in 18 BTIC lines before and after treatment with Sunitinib (1 µM, 6 hours). Transcriptional responses were recorded by Microarray analysis [21]. GSEA Preranked was performed with a pre-ranked list of genes significantly responding to Sunitinib treatment. Accumulation of ATF4 target genes (derived from [36]) at the top of the ranked data set (most highly induced genes) is demonstrated by an enrichment plot. (**B**) Expression of ATF4 mRNA was analyzed by qRT-PCR. Data were normalized to β-Actin and standardized to control treatment (DMSO). The boxplot demonstrates the distribution of relative ATF4 expression after Sunitinib treatment (1 µM, 6 hours) of 18 different BTIC cultures. (**C**) BTICs, U87 and HTZ-349 were cultured and treated with indicated Sunitinib concentrations or Tunicamycin (Tm.) at 0.5 μg/ml. Whole cell protein lysates were prepared 24 hours post treatment and expression of ATF4 was assessed by Western Blot. Actin was used as loading control. (**D**) BTICs, U87 and HTZ-349 cells were exposed to treatment with Sunitinib at indicated concentrations. DMSO was used as an equimolar control. Proliferation was measured after 96 hours by Crystal violet assay. Assays were performed with 5 replicates per condition. Data represent mean ± SD fold changes of expression relative to control treatment.

Our preceding study [21] investigated gene expression and Sunitinib sensitivity in 18 short-term serum-free cultures which originated from different high-grade gliomas enhanced for brain tumor initiating cells (BTIC). The transcriptional responses to short-term treatment were recorded by Microarray analysis. We found an enrichment of ATF4 target genes in the panel of regulated genes with enhanced expression after treatment (Figure 1A). Relative upregulation of the ATF4 mRNA level could be observed for the majority of BTIC cultures (Figure 1B). Since stress-induced ATF4 is mainly regulated on protein level, we performed Western Blot analysis by using a cohort of 6 BTICs. Tunicamycin is an ER-stress inducer which blocks N-linked Glycosylation, a first step of glycoprotein synthesis and was used as a positive control for stress induced ATF4 expression in this study.

Importantly, the ATF4 specific signal was detectable on protein level after low-dose treatment (1 μM Sunitinib) and increased in a dose-dependent manner (Figure 1C, Supplementary Figure 1A).

First, we specified treatment dosages under which a stress related pathway can be attributed a pro-survival role. To analyze the *in vitro* efficacy of Sunitinib on cell proliferation in our BTIC cohort and 2 Glioblastoma immortalized cell lines (U87, HTZ349), we used a range of concentrations which is 50% lower of what is clinically achieved in non CNS tumors (5,1–13,4 µM [22]) (Figure 1D). Molecular characteristics of BTICs and their original tumor tissue are described elsewhere [21, 23, 24].

In 2/6 BTICs (BTIC-6 and 10 respectively) IC50 was not reached. Furthermore, treatment with Sunitinib at a concentration that led to a strong ATF4 signal (2 and 4 μM respectively, Figure 1C) had no significant impact on cell proliferation in 3/6 BTICs (Figure 1D). This suggests that ATF4 rather promotes intracellular homeostasis than pro-death signaling in such conditions. Other reports as well as our own data show that expression and mutation status of targeted receptors are not predictive of response [6, 25] (Supplementary Figure 1B). BTIC-10 was selected for further in-depth analysis since it is characterized by an unreactive response profile and strong ATF4 expression after treatment.

### ATF4 is expressed in human glioblastoma and under starvation conditions *in vitro*

**Figure 2:**
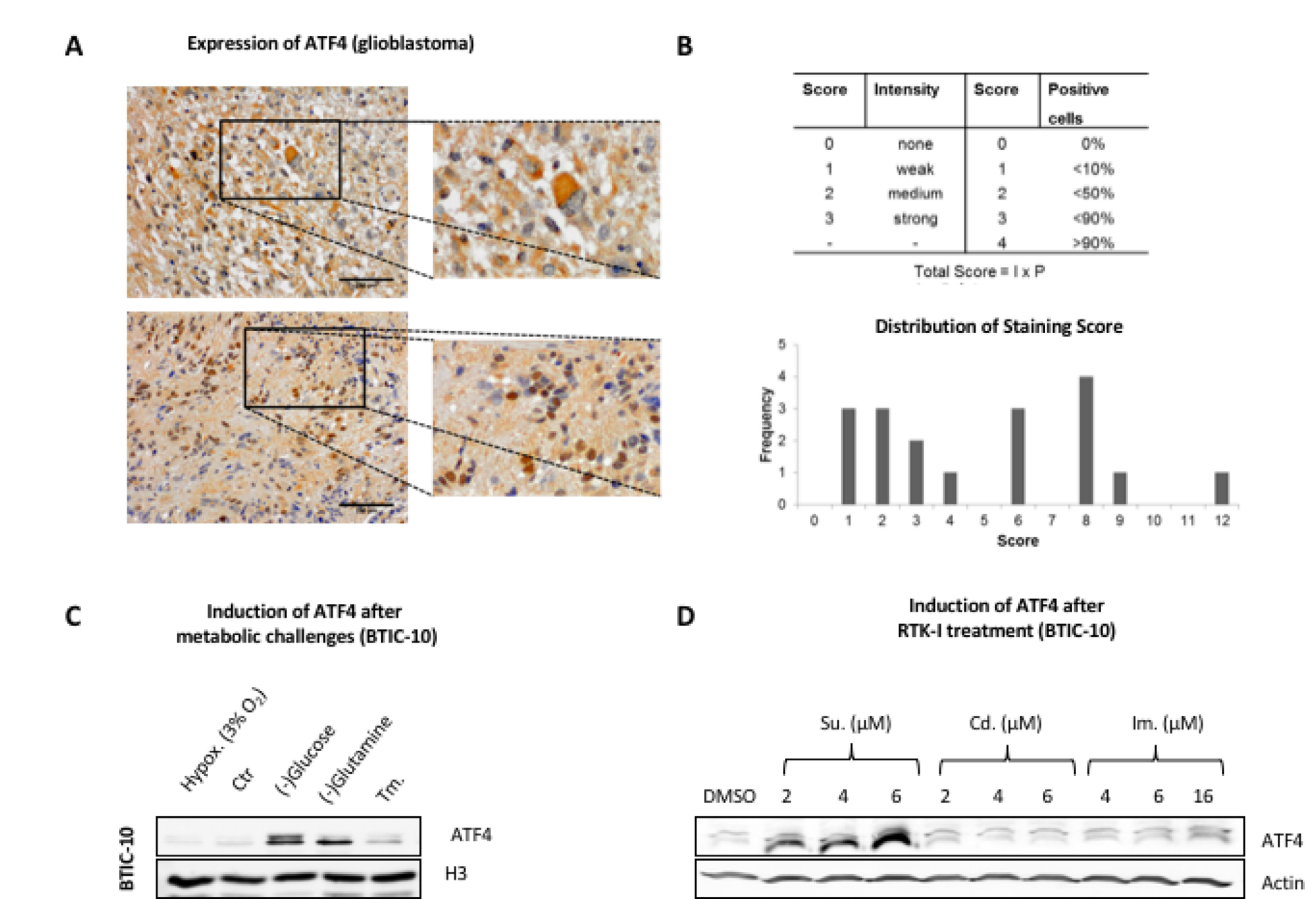
ATF4 protein expression in human resection material and response to metabolic challenges and RTK-Is *in vitro* (**A**) Formaldehyde-fixed paraffin-embedded (FFPE) tissue from resections of human glioblastomas was analyzed for ATF4 expression by immunohistochemistry. Representative stainings are shown. Upper row: cytoplasmatic ATF4 staining pattern. Lower row: nuclear ATF4 staining pattern. (**B**) Quantitative evaluation of ATF4 expression in FFPE tissue (*n* = 18) resections was performed according to the depicted score scheme by three independent investigators. Absolute frequency for each score (ranging from 0–12) is illustrated in the bar graph below. (**C**) BTIC-10 cells were cultured under hypoxic conditions and in glucose- or glutamine-free media or Tunicamycin (Tm.) at 0.5 μg/ml for 48 hours. Nuclear protein extracts were subjected to Western blot analysis. Histon H3 was used as loading control. (**D**) BTIC-10 cells were treated as indicated for 24 hours. Whole protein lysates were subjected to Western Blot analysis. Actin was used as loading control.

ATF4 expression was reported to be increased in hypoxic regions and areas with impaired nutrient supply in several cancer types [13, 15]. To investigate whether the ATF4 mediated stress response also occurs in GBM, we analyzed ATF4 levels in GBM formalin-fixed Paraffin-embedded (FFPE) (*n* = 18) tissue processed under routine conditions at time of diagnosis. Immuno-histochemical stainings showed significant intra- and inter-tumoral variations of ATF4 expression, with ATF4 localized in the nucleus as well as in cytoplasmic regions (Figure 2A, 2B).

Glucose and glutamine were withdrawn from the culture media to mimic nutrient deprivation. The absence of either of them led to ATF4 activation in BTIC-10 (Figure 2C) and BTIC-36 *in vitro* (Supplementary Figure 2A). However, mild hypoxic conditions did not induce the expression of ATF4. Additionally, the effects of Cediranib and Imatinib were tested as alternative small-molecule compounds that target multiple RTKs with PDGFR as a target common to all three RTK-Is [26]. Although the effect on cellular proliferation was similar between Cediranib, Imatinib and Sunitinib (Supplementary Figure 2B) we did not observe any induction of ATF4 except for Sunitinib (Figure 2D). In consistence with protein expression data, significant elevation of ATF4 mRNA was not observed after treatment with Cediranib or Imatinib (Supplementary Figure 2C). However, ATF4 protein expression was detected in U87 and HTZ349 after high dose Imatinib treatment (Supplementary Figure 3A) whereas the lack of glutamine had no effect (Supplementary Figure 2A).

Taken together, our data show that ATF4 protein expression depends on the type of stress as well as on the exposed cell line. The latter finding is consistent with the intra- and inter-tumoral variation of ATF4 expression observed in our Glioblastoma tissue specimens. However, Sunitinib emerged as a potent ATF4-inducer, especially in BTIC-10.

### Sunitinib targets the RTK/PI3K/AKT signaling axis and activates the p-eIF2α/ATF4-pathway

**Figure 3:**
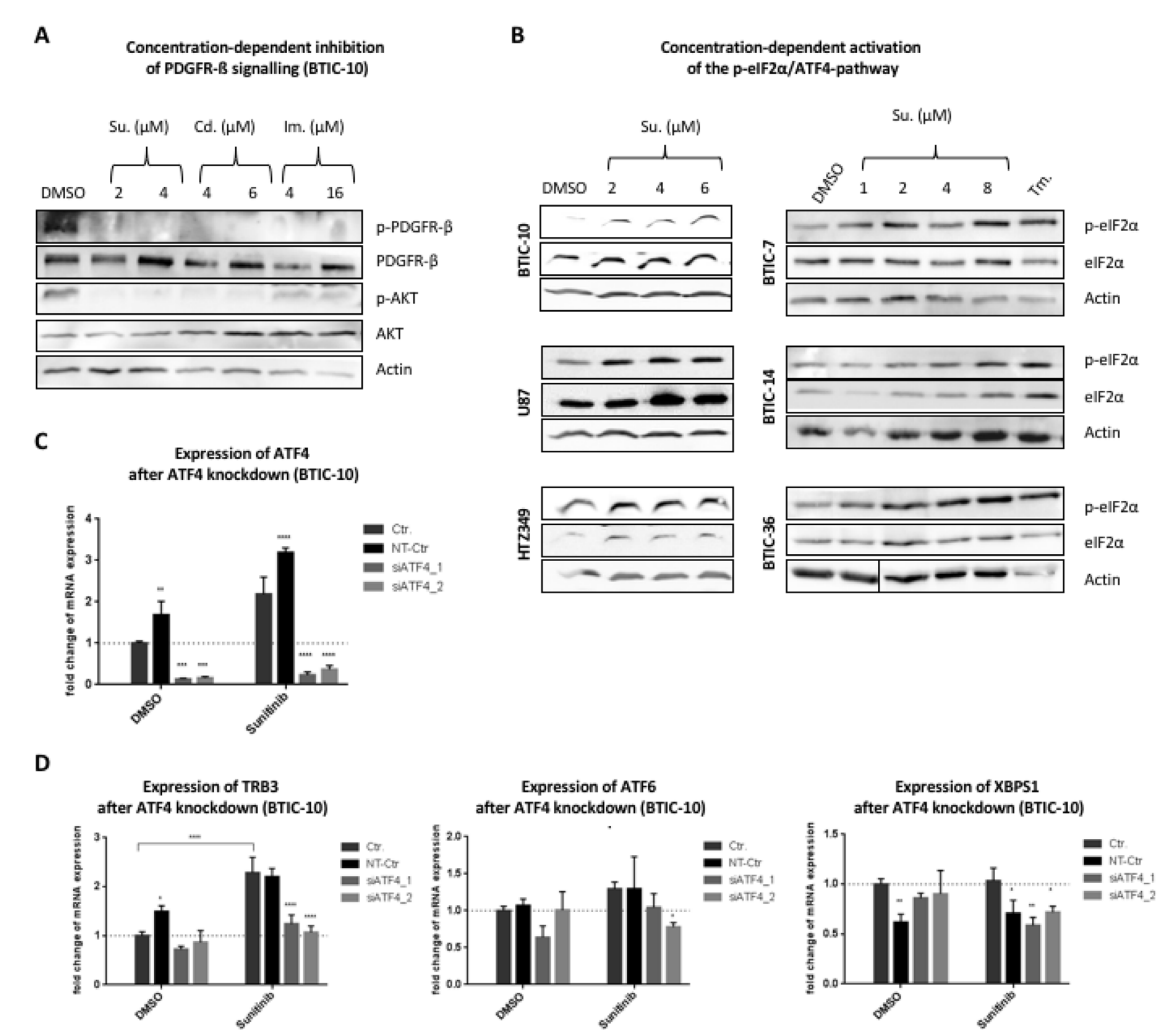
Downstream-signaling after treatment with RTK-Is (**A**) BTIC-10 cells were cultured in growth-factor free media overnight. Sunitinib (Su), Cediranib (Cd), Imantinib (Im) or DMSO (Ctr; equimolar) were added concomitantly with PDGF-AB (25 ng/ml) at the indicated concentrations. After 6 hours, cells were harvested in protein lysis buffer to prepare whole cell lysates. Phosphorylation of PDGFR-β and AKT was assessed by Western Blot. Actin was used as loading control. (**B**) BTICs, U87 and HTZ-349 were cultured and treated with indicated Sunitinib concentrations or Tunicamycin (Tm.) at 0.5 μg/ml. Whole cell protein lysates were prepared 24 hours post treatment and expression of ATF4, eIF2α and phosphorylation of eIF2α was assessed by Western Blot. Actin was used as loading control. (**C**) BTIC-10 cells were transfected with two different siRNAs against ATF4 (siATF4_1/siATF4_2) or non-targeting siRNA (NT-Ctr). Expression of ATF4 mRNA was analyzed by qRT-PCR. (**D**) BTIC-10 was transfected with siRNA against ATF4 or non-targeting siRNA (NT-Ctr) and exposed to treatment with Sunitinib (4 μM). Expression of TRB3, ATF6 and XBPS1 mRNA was analyzed by qRT-PCR. All measurements were performed in triplicates and standard curves were used for relative quantification of expression values. Data represent mean ± SD fold changes of expression relative to control treatment.

In order to confirm that the investigated RTK-Is effectively target the RTK/PI3K/AKT signaling axis under the applied treatment conditions, we exposed BTIC-10 cells to increasing concentrations of Sunitinib, Cediranib and Imatinib. Since all three RTK inhibitors share PDGFR as a common primary target [26], we examined its activation level and the downstream pro-survival kinase AKT by Western Blot (Figure 3A). PDGFR-beta was chosen for analysis since it is widely expressed in our cohort of BTICs, in contrast to PDGFR-alpha (Supplementary Figure 1B). PDGFR phosphorylation was completely abolished with Sunitinib and Cediranib and partly after Imatinib treatment. This indicates that all three RTK-Is are effective in BTIC-10. Again, the Sunitinib dosages applied here were sufficient to abrogate RTK-signaling but had no significant effect on proliferation (Figure 1D).

Since ATF4 protein expression is known to be activated through phosphorylation of eIF2α, we examined eIF2α phosphorylation levels after Sunitinib treatment in a number of BTICs and glioblastoma cell lines (Figure 3B). All investigated cell lines showed an increased phosphorylation signal after exposure to Sunitinib. An increase in eIF2α phosphorylation was again exclusive to Sunitinib when comparing this effect to other RTK-Is (Supplementary Figure 3B). In order to validate that induced ATF4 expression correlates with enhanced transcription factor activity, knockdown experiments were performed. Transfection with siRNA reduced mRNA levels by 85% (Figure 3C). While Sunitinib treatment increased expression of TRB3 (Figure 3D), a well described ATF4 target gene, the effect was attenuated when ATF4 expression was blocked with siRNA (Figure 3D).

The PERK/p-eIF2α axis constitutes one of the three branches of the unfolded protein response (UPR). One of the other include inositol- requiring enzyme 1 (IRE1) which upon activation processes the mRNA encoding unspliced X Box-binding proteins 1 (XBP1u) to produce an active transcription factor, spliced XBP1 (XBP1s). Activating transcription factor 6 (ATF6) is an endoplasmic reticulum (ER) membrane-anchored transcription factor activated by intramembrane proteolysis in the ER stress response [27].

Expression levels of ATF6 and the activated splice-variant of XBP1 (XBP1S), key molecules of UPR pathways that can modulate stress responsive gene expression, were not elevated under ATF4 knock down (Figure 3D). This suggests that stress conditions caused by Sunitinib exclusively activate the p-eIF2α/ATF4 signaling branch (ISR). Taken together, these findings point to a specific role of the ISR in response to Sunitinib.

### The autophagic pathway is activated by sunitinib in BTICs

**Figure 4:**
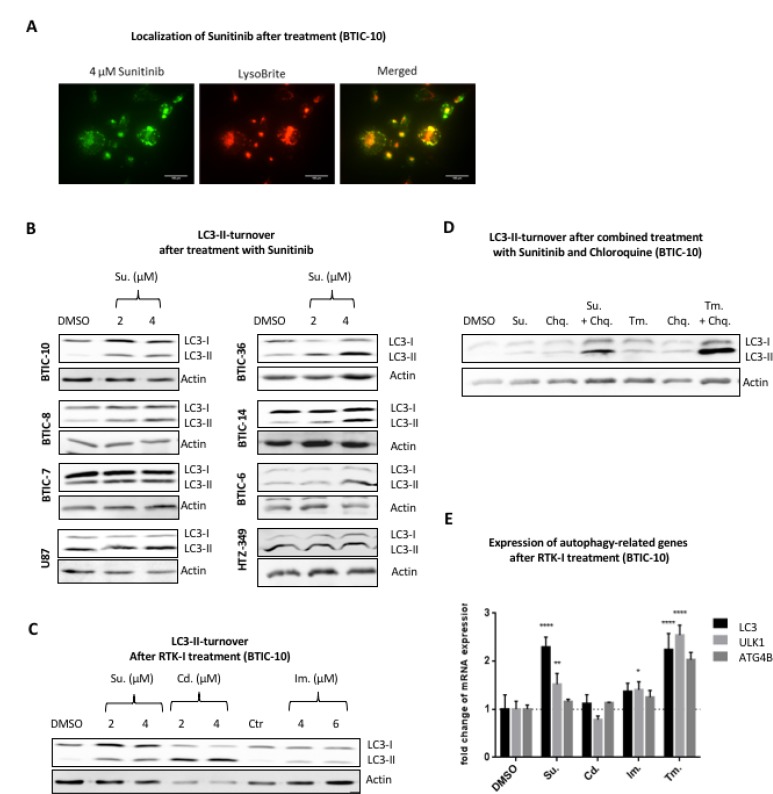
Autophagic turnover after treatment with RTK-I (**A**) Fluorescence images of live BTIC-10 cells treated with 4 μM Sunitinib (green) for 24 hours before adding 1× LysoBrite (red) for 30 min. Sunitinib and LysoBrite were found to be largely co-localized (merged). scale bar = 100 µm. (**B**) BTICs, U87 and HTZ349 were treated as indicated. Whole cell protein lysates were prepared 24 hours post treatment and LC3 expression was assessed by Western Blot. The upper LC3 signal corresponds to the LC3-I isoform and the lower signal to the (autophagosome bound) LC3-II isoform. Actin was used as a loading control. (**C**) BTIC-10 cells were exposed to RTK-Inhibitor treatment with Sunitinib, Cediranib and Imatinib at indicated concentrations. Whole cell protein lysates were prepared 24 hours post treatment and LC3 expression was assessed by Western Blot. The upper LC3 signal corresponds to the LC3-I isoform and the lower signal to the (autophagosome bound) LC3-II isoform. Actin was used as a loading control. (**D**) BTIC-10 cells were exposed to Sunitinib (4 µM), Tunicamycin (Tm., 0.5 µg/ml), Chloroquine (Chq.; 10 µM) or in combination as indicated. Whole cell protein lysates were prepared 24 hours post treatment and LC3 expression was assessed by Western Blot. The upper LC3 signal corresponds to the LC3-I isoform and the lower signal to the (autophagosome bound) LC3-II isoform. Actin was used as a loading control. (**E**) BTIC-10 cells were treated with 4 μM Sunitinib (Su.), 2 μM Cediranib (Ced.), 6 μM Imantinib, 0.25 μg/ml Tunicamycin or DMSO (Control) for 24 hours. Expression of ATF4 was analyzed by qRT-PCR. The measurement was performed in triplicates. A standard curve was used for relative quantification of expression values. GAPDH was used as housekeeping gene. Data represent mean ± SD fold changes of expression relative to control treatment.

Because of the auto-fluorescent properties of Sunitinib, its intracellular distribution can be monitored in live cells. We found that Sunitinib co-localized with the lysosomal marker LysoBrite in BTICs (Figure 4A). An entrapment of Sunitinib in lysosomes has been reported by several other studies [22, 28] and refers to a mechanism specified as lysosomal drug sequestration where hydrophobic weak base drugs accumulate in the acidic lysosomal lumen. Hence, we speculated that Sunitinib leads to an inhibition of the autophagic degradation process by raising the lysosomal pH. Autophagy can be followed in cells by monitoring the processing of microtubule-associated protein 1 (MAP1) light chain 3β (MAP1LC3B) [29]. MAP1LC3B is produced in a pro-form (pro-MAP1LC3B), which is cleaved by ATG4B into a cytosolic form (referred to as LC3-I) during initiation of autophagy. LC3-I is conjugated to the lipid phosphatidylethanolamine (referred to as LC3-II), which is then inserted into the membrane of the growing autophagic vesicle [30].

We determined the expression and processing of LC3 after *in vitro* treatment. As expected, with Sunitinib treatment, the conversion of LC3 from its cytosolic form (LC3-I) to its lipidated membrane-bound form (LC3-II) was highly enhanced in BTIC-10 and other BTICs (Figure 4B) supporting the hypothesis of autophagosome accumulation. Cediranib and Imatinib are also hydrophobic weak base drugs and consequently lead to enhanced LC3-II conversion with Cediranib exhibiting the strongest effect (Figure 4C). However, LC3-II accumulation was not observed in glioblastoma cell lines U87 and HTZ-349 by RTK-I treatment (Figure 4B, Supplementary Figure 4A, 4B). Importantly, Sunitinib also increased LC3-I level in BTIC-10 (Figure 4B). To further investigate a direct activation of autophagy by Sunitinib we simultaneously treated BTIC-10 with Cholorquine. Chloroquine is also a lysomotropic agent and is therefore commonly used as an autophagy inhibitor. The combination of both drugs additively increased the LC3-II signal which supports the assumption that Sunitinib also promotes autophagy initiation (Figure 4D).

To investigate if this observation might indicate enhanced transcription of autophagy-related genes, we performed qRT-PCR analysis. Elevated mRNA levels of LC3, ULK1 and, to a lesser extent, ATG4B were found in response to Sunitinib (Figure 4E). ULK1 is a structural part of the ULK1/2 kinase and a key initiator of the autophagic process. At the same time, no significant change of LC3 was observed after Cediranib or Imatinib treatment. Furthermore, mTOR activation is not reduced after Sunitinib (data not shown), indicating that direct autophagy activation is independent of RTK/PI3K/AKT signaling in this context.

In summary, our data show that Sunitinib concomitantly decreases autophagic clearance due to lysosomal drug sequestration and directly activates autophagy by enhanced expression of autophagy-related genes.

### ATF4 is involved in sunitinib induced autophagy activation

In order to examine a causal link between activation of ISR and autophagy, BTIC-10 and BTIC-36 were treated with siRNAs against ATF4.

**Figure 5:**
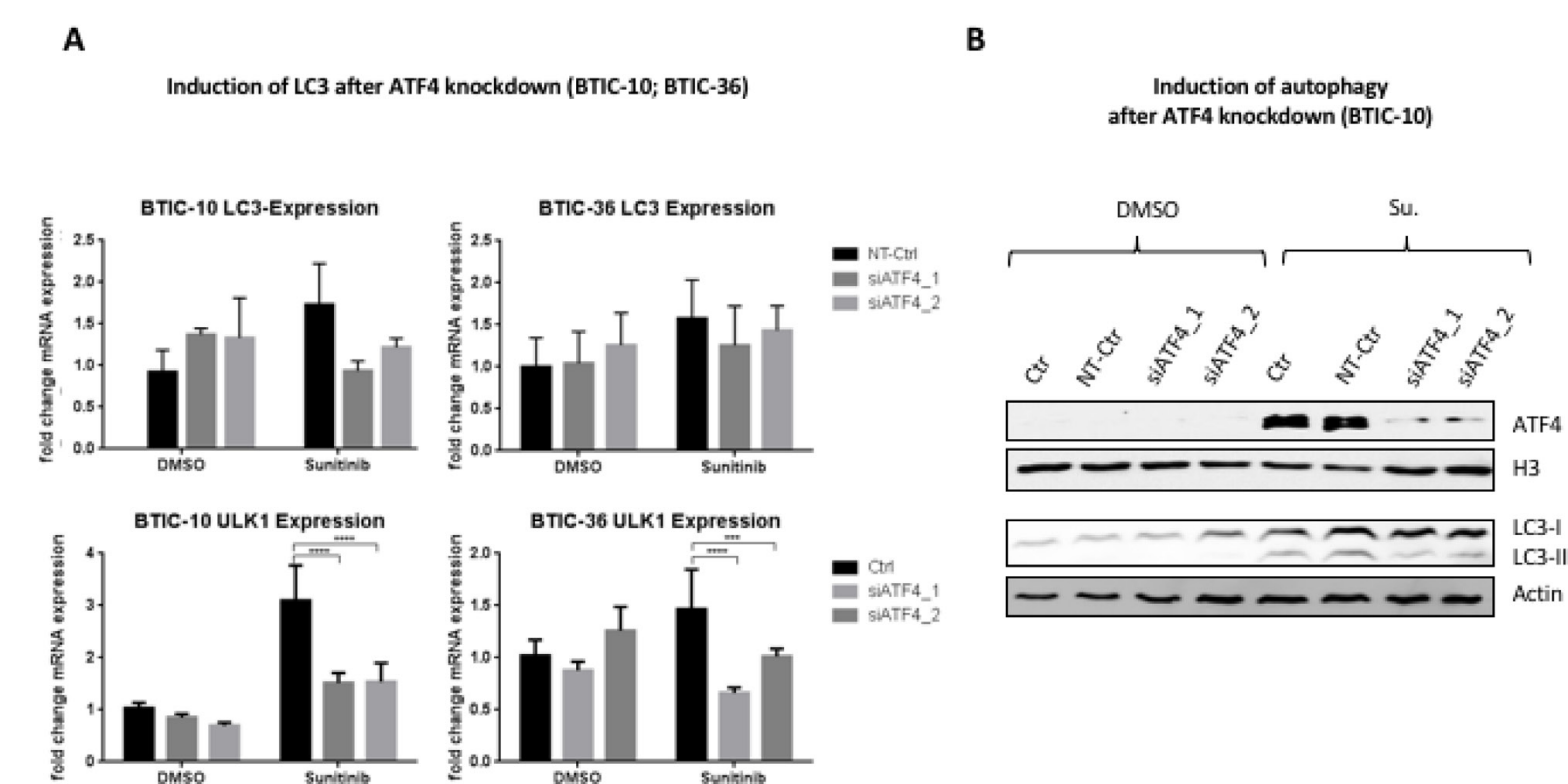
Influence of ATF4-knockdown on sunitinib induced autophagy (**A**) BTIC-10 and BTIC-36 cells were transfected with ATF4 siRNAs or non-targeting siRNA (NT-Ctr) followed by treatment with 4 μM Sunitinib or DMSO (Ctr) for 24 hours. LC3 expression and ULK1 expression was analyzed by qRT-PCR. The measurement was performed in triplicates. A standard curve was used for relative quantification of expression values. Data represent mean ±SD fold changes of expression relative to control transfection with NT-Ctr siRNA. (**B**) BTIC-10 cells were transfected with ATF4 siRNAs or non-targeting siRNA (NT-Ctr) followed by treatment with 4 μM Sunitinib or DMSO (Ctr) for 24 hours. Nuclear protein extracts were used to analyze ATF4 expression by Western Blot. (Histone) H3 was used as a loading control. Whole cell protein extracts were subjected to Western Blot analysis to detect LC3-I isoform (upper band) and LC3-II isoform (lower band).

Sunitinib induced upregulation of ULK1 significantly decreased when BTICs were depleted of ATF4 by RNA interference (RNAi) (Figure 5A). Additionally, albeit not statistically significant, LC3 mRNA upregulation was reduced, and LC3-I to LC3-II conversion was partially abrogated in Sunitinib treated BTIC-10 with ATF4 knock-down (Figure 5A, 5B). This residual LC3-II signal after Sunitinib treatment and concomitant ATF4 knockdown could be explained by the observed Sunitinib-induced inhibition of autophagosome degradation by accumulating in acidic vesicles (Figure 4A).

### ATF4 increases insensitivity to treatment in BTIC-10 cells

**Figure 6:**
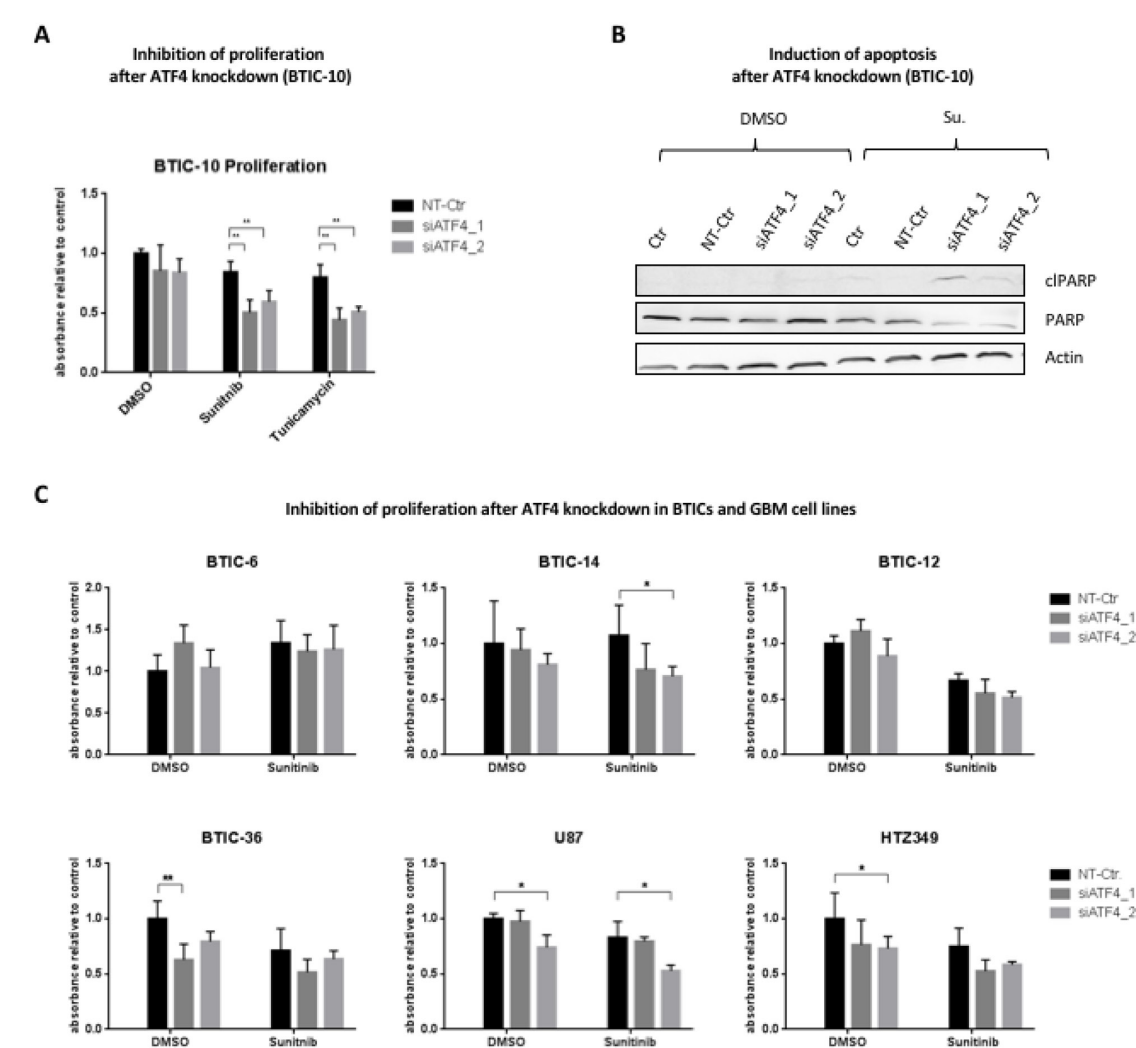
Influence of ATF4 knockdown on proliferation and apoptosis (**A**) BTIC-10 cells were transfected with siRNAs against ATF4 or non-targeting siRNA (NT-Ctr) and exposed to treatment with Sunitinib (4 μM), Tunicamycin (0.25 μg/ml) or DMSO as control. Proliferation was measured by Crystal Violet assay 48 hours after incubation. Data represent mean ± SD fold changes of expression relative to control transfection with NT-Ctr siRNA. (**B**) BTIC-10 cells were transfected with siRNAs against ATF4 or non-targeting siRNA (NT-Ctr) and exposed to treatment with Sunitinib (4 μM) or DMSO (ctr) for 24 hours. Whole cell protein lysates were subjected to Western blot analysis. Immunostaining against the PARP cleavage product (cl-PARP) was performed to assess apoptosis. Actin was used as a loading control. (**C**) BTICs were transfected with siRNAs against ATF4 or non-targeting siRNA (NT-Ctr) and exposed to treatment with Sunitinib (4 μM) or DMSO as control at equimolar concentration. Proliferation was assessed by Crystal Violet assay 48 hours after incubation. Data represent mean ± SD fold changes of expression relative to control treatment transfected with NT-Ctr siRNA.

To further detail the mechanism of ATF4 in BTIC-10, we investigated proliferation and apoptosis in cells transfected with siRNAs targeting ATF4 and exposed to Sunitinib (Figure 6A, 6B). In BTIC-10, ATF4 knockdown decreased survival by about 30% compared to controls. Consistently, we observed an increased cleavage of poly (ADP-ribose) polymerase (PARP) by immunoblotting in BTIC-10 (Figure 6B) and BTIC-36 (Supplementary Figure 5A) that serves as an indicator for elevated apoptosis. These results support the hypothesis that ATF4 has a protective role in response to Sunitinib. Of note, no cleavage of effector caspases-3 and -7 was observed (Supplementary Figure 5B). We analyzed the impact of ATF4 knockdown on cell growth and Sunitinib treatment effects with an extended panel of BTICs and glioblastoma cell lines (Figure 6C). Overall, the effects varied among BTICs as well as cell lines. BTIC-14 showed a similar effect compared to BTIC-10 whereas for BTIC-36, U87 and HTZ349, ATF4 knockdown also impaired cell growth in control conditions. For BTIC-6 and BTIC-12, ATF4 knockdown had no effect on cell growth independent of the applied treatment condition. Hence, the function and significance of ATF4 and consequently the ISR is highly cell-line and treatment specific. A pro-survival role of ATF4 under physiologically relevant dosage of Sunitinib was confirmed in BTIC-10.

## DISCUSSION

Receptor tyrosine kinases (RTK) are promising therapeutic targets and RTK inhibitors (RTK-I) have been approved in a number of tumors. However, all clinical trials in glioblastoma (GBM) attempting to establish any clinical benefit of RTK-I have failed so far. Point mutations within the kinase domain, modifications of signaling pathways, or altered drug flux have been implicated in drug resistance [31]. In addition, eukaryotic cells respond and adapt to micro-environmental stressors by adopting signal transduction pathways that regulate the adaptive and protective phenotype [32].

In this study, we observed an activation of ATF4 primarily in response to the RTK-I Sunitinib. ATF4 expression on protein levels correlated with the applied Sunitinib concentration. Although ATF4 is known to activate pro-apoptotic factors [33], previous reports have established that failure to fully induce ISR by the eIF2alpha kinases PERK and GCN2 and hence to activate ATF4 reduces tumor cell growth *in vitro* and *in vivo* [15, 34, 35]. Downregulation of ATF4 has further been shown to prevent cancer cells from being resistant to anticancer drugs [36], indicating that ATF4 expression is required for cancer cell survival in response to chemotherapy. In line with these data, Adjibade *et al.* have shown that minimal expression of ATF4 in stress granule-forming hepatocarcinoma cells is relevant for their survival under RTK-I Sorafenib [37]. However, Weatherbee *et al.* demonstrated that during simultaneous treatment of GBM cell lines with Temozolomide and JLK1486, a novel ER-stress inducing agent, the initial cytoprotective mechanism becomes cytotoxic due to prolonged ER-stress [38]. Although we did not test Sorafenib, ATF4 expression was hardly detectable in RTK-Is other than Sunitinib in this study.

McTigue and colleagues studied the affinities of RTK-Is to a panel of 317 kinases and found that Sunitinib has a low selectivity for specific tyrosine kinases [39]. Therefore, it seems plausible that off-target effects unique to Sunitinib lead to discrete intracellular responses. Mitochondrial dysfunction and inhibition of AMP-activated protein kinase (AMPK) have been reported in association with Sunitinib induced toxicities [40, 41]. At high cellular energy levels, AMPK remains in an inactive state to minimize ATP production through catabolic pathways. Preliminary data confirmed inhibition of AMPK in Sunitinib treated BTICs (data not shown). A misinterpreted energy level could lead to starvation and consequently ATF4 expression. Nevertheless, further studies are required to unambiguously identify upstream molecular events that lead to activation of the eIF2α/ATF4 pathway. Resolving the upstream molecular events that lead to eIF2α/ATF4 activation under Sunitinib may also provide an approach to resolve the heterogeneous results that were observed after ATF4 knockdown.

In addition to off-target effects, physico-chemical properties of a drug are also critical for its intracellular action. Most RTK-Is are hydrophobic weak bases that facilitate breaching the plasma membrane and other intracellular membranes [42]. RTK-Is usually behave as lysomotropic agents (late-stage autophagy inhibitors), meaning that they accumulate in acidic intracellular organelles [43]. It has been reported that acidic lysosomes may be involved in resistance through intracellular sequestration of Sunitinib due to its chemical characteristics [22]. Indeed, we found co-localization of acidic vesicles and Sunitinib, likely leading to a reduced availability of Sunitinib in the cytoplasm. Additionally, by raising the vesicular pH, the lysosomal degradation step is inhibited, leading to an enhanced LC3-II signal, as seen in our assays. We propose that this mechanism of resistance applies to other agents as well, including RTK-Is. In fact, we observed an increase of the LC3-II to LC3-I ratio also after treatment with Cediranib and Imatinib.

In addition, ATF4 knockdown resulted in an elevated level of Poly (ADP-ribose)-Polymerase 1 (PARP) cleavage product (89kDa), pointing to an increase in apoptosis. Interestingly, no cleavage of caspases-3 and -7, known to be upstream effectors of PARP cleavage, was observed. Cleavage of PARP can also be mediated independent of caspases by cathepsins, which belong to the family of lysosomal proteases. Those can be released when the lysosomal pH is lowered, as e.g. by application of lysosomotropic agents [44]. RTK-Is Imatinib and Sorafenib are known inducers of lysosome-dependent cell death [45]. Our results indicate that a similar mechanism might be relevant for Sunitinib.

Autophagy activation during treatment with RTK inhibitors has been commonly observed as an obstacle to more efficacious therapy and has been associated with the limited efficacy of RTK inhibitors [20]. These findings provide a rationale for a combined RTK/autophagy inhibitor treatment. Indeed, several studies suggest that targeting autophagy sensitizes glioma cells to treatment [44, 46]. Lobo and colleagues reported on the combined efficacy of Cediranib and the late-stage autophagy inhibitor Quinacrine in glioma cells *in vitro* [47]. There, autophagy was exclusively monitored by LC3-II accumulation. One has to keep in mind that this does not allow unraveling autophagic flux from autophagic degradation blockage. In our study, we showed that Sunitinib, but not Cediranib or Imatinib, induced LC3 and ULK1 gene expression in BTICs.

For hypoxia, several studies demonstrated that transcriptional regulation of MAP1LC3B is a rate-limiting step for the induction of autophagy [10, 14]. As ATF4, LC3 and ULK1 expression is concomitantly and solely activated after exposure to Sunitinib in BTIC-10, a causal relationship appears obvious. In fact, knockdown of ATF4 in BTIC-10 led to downregulation of ULK1, partly reduced LC3 expression and LC3-I to LC3-II conversion. In line with our results, Pike *et al.* have shown that ATF4 transcriptionally upregulates ULK1 expression [48]. Thus, our data suggest that only Sunitinib induces autophagic flux beside its lysosomal entrapment, via ATF4. Further in-depth analyses which, among others, take kinetic characteristics into account are needed to confirm this assumption.

The PI3K/AKT/mTOR pathway is one of the most important signaling pathways that regulate autophagy [49], and at the same time represents one of many downstream pathways activated by RTKs. On a functional level, we confirmed and specified the efficacy of the selected receptor tyrosine kinase inhibitors (RTK-Is) *in vitro,* corresponding very well to findings of other studies [5, 47].

Molecular and functional responses are heterogeneous within our BTIC and glioblastoma cell lines. BTICs were reported to mirror both the genotype and the transcriptome more closely and stably than conventional glioma cell lines [50, 51]. However, numerous studies–including our own - still utilize conventional cell lines for the investigation of pathogenic pathways in order to identify and validate therapeutic targets. To evaluate the transferability of results, we analyzed molecular and functional differences in response to treatment and stress conditions in several BTICs as well as U87 and HTZ-349. We found that all RTK-Is tested were effective in BTICs, but to a lesser extent in the established cell lines. Although BTICs are known to be more resistant to classical chemotherapeutics like Temozolomide and radiation therapy [38, 39], our results indicate that this does not apply to RTK-Is. Interestingly, ATF4 knockdown in U87 did not have any influence on LC3-I or LC3-II levels irrespective of treatment. Considering the impact on cells that were not exposed to any additional stress, ATF4 deprivation did not alter any treatment induced effects in U87 on a functional level. These results also apply to HTZ-349 and BTICs apart from BTIC-10. So far, our data therefore indicate that the impact of stress activated pathways to compensate for micro-environmental or treatment induced perturbations varies greatly among different cells. We assume that supplemented FCS could induce a set of multiple RTK-signaling cascades compared to stem cell media. This could in turn lead to signaling redundancy, where other members of a protein family or distinct signaling molecules can compensate for the inhibited component to maintain the activity of key downstream circuits even in the presence of drugs.

Taken together, our results clearly demonstrate that resistance and adaption mechanisms are unique to individual genetic and/or molecular backgrounds of BTIC or cell lines, are highly context-dependent, and even highly vary among members of the same compound class. The selection of the appropriate *in vitro* model is of great importance and can lead to opposing results as demonstrated by our study. A better understanding of these aspects may aid the design of next generation compounds and may also lead to effective drug combinations for GBM therapy. Our work therefore underscores the need for a more individualized analysis of patient-derived tissue before therapy.

## MATERIALS AND METHODS

### Cell culture and treatment conditions

Native brain tumor tissue samples from human glioblastomas were obtained from patients undergoing surgical resection at the local Department of Neurosurgery [14]. All tumors were histologically classified according to the 2007 WHO classification of tumors of the central nervous system by the local neuropathologist (MJR). The ethics committee of the University of Regensburg, Regensburg, Germany (No° 11-103-0182) approved the study and all patients gave written informed consent.

Molecular characterization of tumor samples and corresponding *in vitro* cell culture was performed as described earlier [14]. Briefly, BTIC-6, -10, -12, -14, and -36 were grown in stem-cell permissive medium (RHB-A, Stem Cell Sciences, Cambridge, UK) supplemented with EGF/bFGF (20 ng/ml each). The human high-grade glioma cell line U87MG was obtained from American Type Culture Collection (ATCC, Manassas, VA, USA). The cell line named ‘HTZ-349’ was a primary tumor cell culture derived from human glioblastoma as described before [40]. Growth medium for U87 and HTZ-349 was Dulbecco’s modified Eagle’s medium (Invitrogen, Carlsbad, CA, USA) supplemented with 10% fetal calf serum (FCS).

All cell lines were maintained as standard monolayer or sphere cultures at 37° C, 5% CO2, 95% humidity in a standard tissue culture incubator. For hypoxia experiments, cells were incubated in a hypoxia incubator (Heraeus, Hanau, Germany) at 3% O_2_. Glucose and glutamine deprivation was achieved by culturing cells in respective media (DMEM, no glucose; advanced DMEM, no glutamine; Thermo Fisher Scientific, Waltham, MA, USA). Sunitinib (Sigma Aldrich, St. Louis, Missouri, USA) and Cediranib (Selleckchem, Houston, USA) were diluted as 5 mmol/l stock solutions in DMSO. For Imatinib and Tunicamycin (Sigma Aldrich, St. Louis, Missouri, USA), autoclaved H_2_O was used as solvent to prepare stocks of 5 mmol/l and 1.7 mg/ml, respectively. Stock solutions were added to the cell culture media immediately before treatment and in quantities that were needed to reach the required final concentration. Equal concentrations of DMSO were applied to all samples (including controls) to mask any DMSO induced effect. Staurosporine (Sigma Aldrich, St. Louis, Missouri, USA) was added at a final concentration of 1 µM.

### Transient transfection

Cells were plated in 6-well plates 24 hours before transfection at a density of 70–80%. The following double-stranded RNA oligonucleotides were generated and purchased from Eurofins Genomics: 5′- CAGAUUGGAUGUUGGAGAA-3′ for siATF4_1; 5′- GAGAUAGGA AGCCAGACUA-3′ for siATF4_2; 5′- UGGUUUACAUGUCGACUAA-3′ for NT-Ctr.

Transient transfections were performed according to manufacturer’s instructions (Invitrogen) with modifications. Lipofectamine 3000 (Invitrogen) was diluted in Opti-MEM medium (Invitrogen) at 7.5 μl/ml. Likewise, 300 pmol siRNA were added to 1 ml Opti-MEM medium respectively. Both mixtures were incubated separately for 5 min at room temperature before they were combined and incubated for another 20 min. The transfection cocktail was diluted 1:3 in antibiotic- (and FCS-) free culture medium and added on top of the cells. The medium containing the transfection reagents was removed after 24 hours and substituted by the customary media together with treatment if applicable.

### Crystal violet proliferation assay

2,500 cells per well were plated in 100 µl of medium in 96-well flat bottom plates. After subsequent transfection and/or treatment, medium was removed and 50 µl of crystal violet solution (0.5% crystal violet, 20% methanol) were added to each well. After a 10-minute incubation time, crystal violet was removed by submerging the plates in water 3-5 times. To remove excess water, plates were robustly tapped on paper towels and subsequently left to dry overnight. Once dry, 50 µl of Sodium citrate solution (0.1 M Sodium Citrate, 50% ethanol) were added to each well and absorbance was measured photometrically at 550 nm.

### Protein extraction and Western blot analysis

For whole cell protein lysates, cells were lysed in RIPA buffer (50 mM Tris, 150 mM NaCl, 0.5% Triton X100, 0.5% Deoxycholate, 4-(2-Aminoethyl)benzenesulfonyl fluoride hydrochloride, Halt™ Protease Inhibitor Cocktail; Thermo Fisher Scientific, Waltham, MA, USA).

For preparation of cytoplasmic and nuclear fractions, cytoplasmic extraction buffer (10 mM Tris (pH 8), 1 mM EDTA (pH 8), 150 mM NaCl, 0.1% IGEPAL) was added to the cell culture, and cells were scraped using a cell scraper, vortexed, and centrifuged. The supernatant containing cytoplasmic proteins was carefully removed, and the cell pellet containing the nuclear fraction was resuspended in 50 µl of nuclear extraction buffer (10mM Tris (pH 8), 1 mM EDTA (pH 8), 400 mM NaCl). Genomic DNA was cleared by centrifugation at 15.000 rpm for 15 min. Protein solutions were stored at −80° C.

For Western blot analysis, 15 μg of whole cell protein lysates, 10 μg of nuclear protein lysates and 15 μg of cytoplasmic protein lysates were used. Detailed gel electrophoresis and Western Blot procedures are described elsewhere [14]. Primary antibodies used were: Rabbit anti-phospho AKT, (Ser473), rabbit anti-AKT, rabbit anti-phospho eIF2α, rabbit anti-eIF2α, rabbit anti-cleaved PARP, rabbit anti-PARP, rabbit anti-Histone H3, rabbit anti-phospho PDGFR-β, rabbit anti-PDGFR-β, (all purchased from Cell Signaling, Danvers, USA), rabbit anti-ATF4 (Santa Cruz Biotechnology, Heidelberg, Germany), rabbit anti-β-Actin (Sigma Aldrich, Missouri, USA), and LC3 (Novus, Littleton, USA).

### RNA isolation and analysis of gene expression by real time RT-PCR

RNA isolation and cDNA generation were performed according to standard protocols described before [14]. The following primers were used for semiquantitative real-time PCR analysis: ACTIN, rev AGAGGCGTACAGGGATAGCA, fwd TGGC ACCACACCTTCTAC AAT; ATF4, rev GTCTGGT TATCTCCTTCA, fwd CCCTTCACCTTCTTAAACCT; ATF6, rev TCGGAGGTAAGGAGGAACTGACG, fwd CCGCAGAAGGGGAGACAC; GAPDH, rev ACCCTG TTGCTGTAGCCA, fwd CCACTCCTCCACCTTTGAC; LC3, rev CTGTGTCCGTTCACCAACAG, fwd AGCAG CATC CAACCAAAATC; XBP1S, rev GCTGGCAGGCT CTGGGGAAG, fwd TGCTGAGTCCGCAGCAGGTG; ULK1, rev CGTCTGAGACTTGGCGAGGT, fwd TCGA GTTCTCCCGCAAGG; ATG4B, rev AGTATCCAAACG GGCTCTG, fwd ACTGGGAAGATGGACGCAG; RPLP0, rev GATGGATCAGCCAAGAAGGC, fwd CTGTCTGC AGAT TGG CTACCC.

Quantification of mRNA expression was performed by real-time PCR (Mx3000P Quantitative PCR [qPCR] System, Stratagene) based on SYBR-Green I fluorescence (Brilliant III Ultra Fast SYBR GREEN QPCR Master Mix, Agilent Technologies, 600883). cDNA samples of BTICs were diluted 1:10. All samples were used in triplicates. For each reaction, melting curves were used to verify the identity of the amplification products. Three serial fivefold dilutions of cDNA, a mixture of all used cDNA-samples, were amplified in duplicates to construct standard curves for both the target gene and the reference (GAPDH, RPLP0). Alternatively, relative expression changes after treatment were quantified using the ΔΔCT-method.

### Subcellular co-localization studies

Staining of cells with LysoBrite™ DeepRed (Biomol GmbH, Hamburg, Germany) was performed according to the manufacturer´s instructions with minor modifications. Briefly, medium was removed and cells were preincubated with 4 µM Sunitinib in PBS for 30 min at 37° C. LysoBrite was added to a 1× final concentration and cells were incubated for another 30 min at 37° C. The staining solution was then aspirated, cells were washed twice with PBS and examined in PBS under the microscope (Zeiss Mikroskop Axio Observer.Z1). No fixation was performed since Sunitinib fluorescence thereby was greatly reduced or lost.

### Immunohistochemistry

Immunohistochemical staining was performed following a standard protocol [50]. Briefly, 5-µm sections were cut, and slides were deparaffinized. Then sodium citrate buffer antigen retrieval (30 min) and blocking was performed. Slides were incubated with the primary anti-ATF4 antibody (sc-200; Santa Cruz Biotechnlogy, Dallas, Texas; 1:100 dilution) for 30 minutes. The EnVision™+ Dual Link System-HRP (Dako by Agilent Technologies, Santa Clara, CA) was used for detection of antibody binding according to the manufacturer’s protocol. Finally, nuclei in the immunostained sections were counterstained with haematoxylin.

### Gene enrichment and functional association analysis using microarray expression data

GSEA was performed on a gene list ranked by Sunitinib induced gene expression changes which were obtained by microarray analysis in a previous study ([21]; GEO accession: GSE51305). A predefined gene set consisting of genes regulated by ATF4 knockdown were derived from the Molecular Signature Database (MSigDB) (Genset name: IGARASHI_ATF4_TARGETS_DN; source publication: [36]); The correlation between the ranked gene list and the ATF4 related geneset was analyzed by the GSEA preranked tool (http://software.broadinstitute.org/gsea/downloads.jsp.; [51–53]).

### Statistical analysis

Graph Pad Prism version 6.0 was used to calculate a one-way ANOVA to compare the results (mean values and SDs) of control vs. treated cells. Dunnett’s post-hoc test was used to control for multiple comparisons. The level of significance was set at ^*^P < 0.05, ^**^P < 0.01, ^***^P < 0.001 and ^****^P < 0.0001

## SUPPLEMENTARY MATERIALS FIGURES


