# Supplementary material for: ATF4 contributes to autophagy and survival in sunitinib treated brain tumor initiating cells (BTICs)
Source: Oncotarget. 2019 Jan 8;10(3):368–82. doi: 10.18632/oncotarget.26569 (PMC6349458; doi:10.18632/oncotarget.26569)
Supplement: Supplementary file 1 [file oncotarget-10-368-s001.pdf]

ATF4 contributes to autophagy and survival in sunitinib treated brain tumor initiating cells (BTICs)

SUPPLEMENTARY MATERIALS

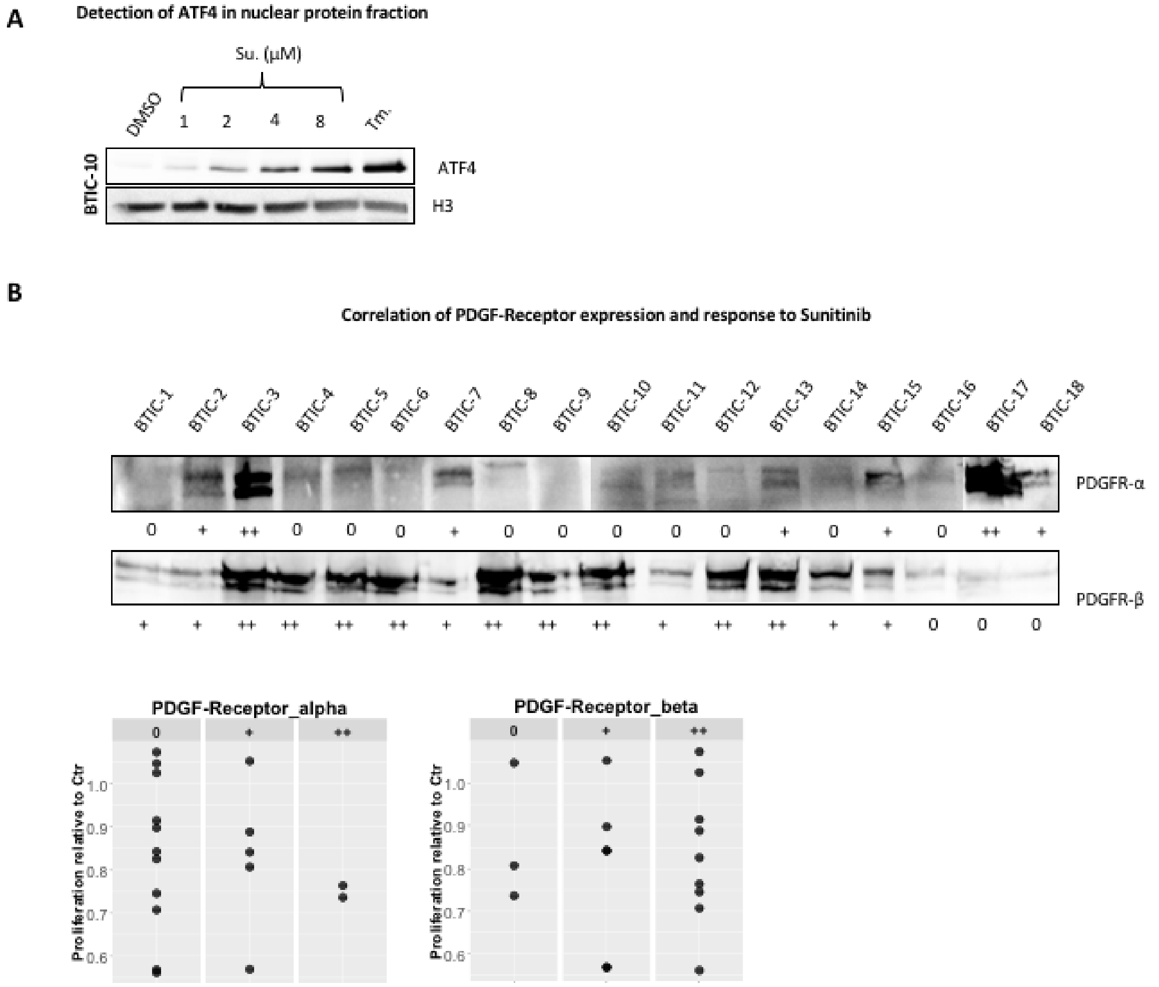

Supplementary Figure 1: ATF4 expression in BTIC-10, semi-quantitative PDGF receptor-α/β expression in BTICs. (**A**) BTIC-10 cells were treated with Sunitinib at indicated concentrations or Tunicamycin (Tm.) at 0.5 µg/ml for 24 hours. Nuclear protein extracts were subjected to Western Blot analysis. Histon H3 was used as loading control. (**B**) Whole cell protein lysates were prepared from 18 BTIC cultures. For Western Blot analysis 30 µg of total protein/BTIC culture were used. Expression intensity of PDGF Receptor-α and PDGF Receptor-β were graded as follows by three independent investigators: ++ strong, + moderate, 0 not expressed. Intensities of PDGF Receptor were plotted against proliferation after Sunitinib treatment (1 µM) for 18 BTICs. Proliferation after Sunitinib treatment was normalized to the respective control (DMSO) and published elsewhere [21].

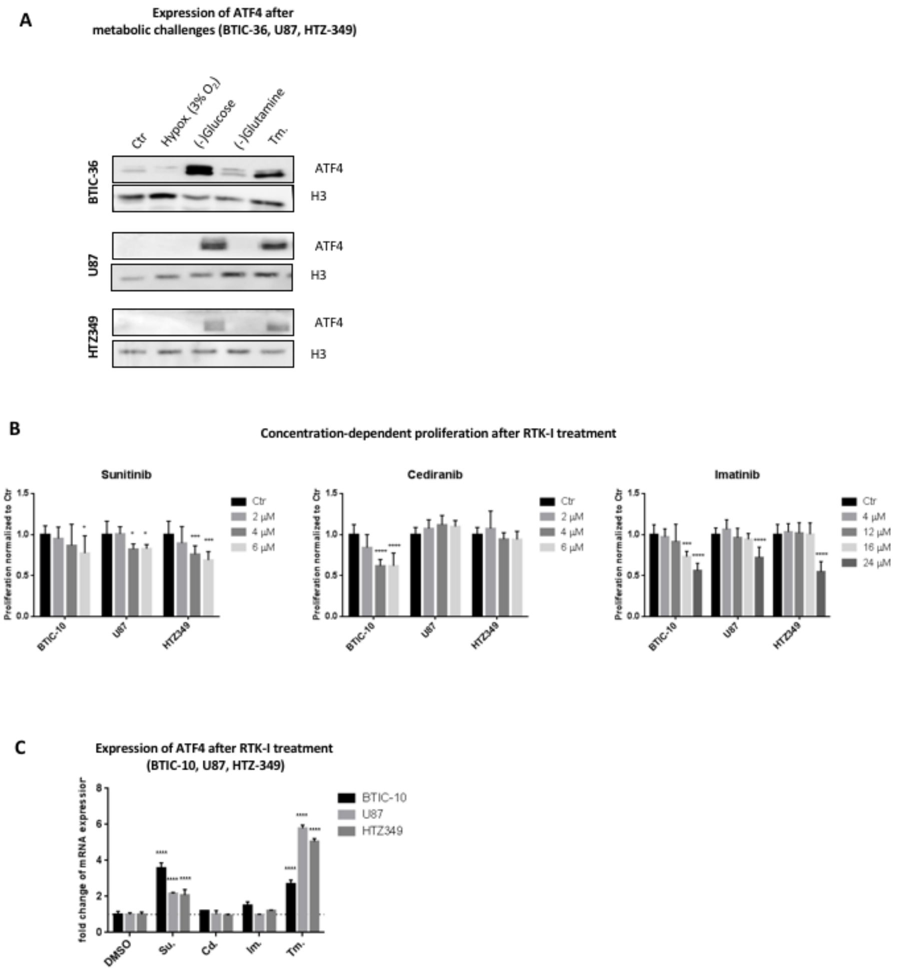

Supplementary Figure 2: ATF4 expression and cellular proliferation in response to micro-environmental stress factors and RTK-I treatment in BTICs and GBM cell lines. (**A**) BTIC-36, U87 and HTZ-349 cells were cultured under hypoxic conditions and in glucose- or glutamine-free media or Tunicamycin (Tm.) at 0.5 µg/ml for 48 hours. Nuclear protein extracts were subjected to Western Blot analysis. Histon H3 was used as loading control. (**B**) BTIC-10, U87 and HTZ-349 cells were exposed to treatment with Sunitinib, Cediranib and Imatinib at indicated concentrations. DMSO was used as an equimolar control. Proliferation was measured after 96 hours by Crystal violet assay. Assays were performed with 5 replicates per condition. Data represent mean ± SD fold changes of expression relative to control treatment. (**C**) BTIC-10, U87 and HTZ-349 cells were treated with 4 µM Sunitinib (Su.), 2 µM Cediranib (Ced.), 6 µM Imantinib (Im.), 0.25 µg/ml Tunicamycin or DMSO (control) for 24 hours. Expression of ATF4 was analyzed by qRT-PCR. The measurement was performed in triplicates. A standard curve was used for relative quantification of expression values. Data represent mean ± SD fold changes of expression relative to control treatment.

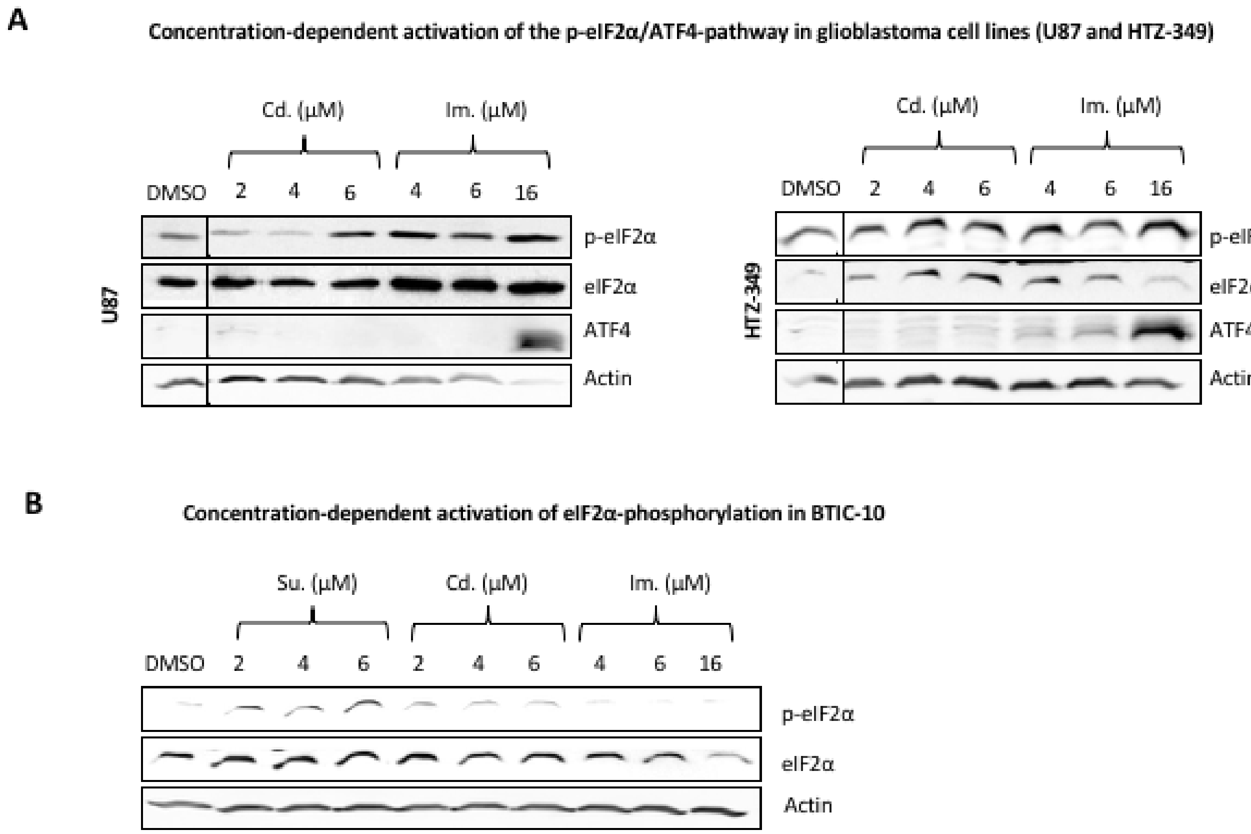

Supplementary Figure 3: ATF4 expression and signaling pathway induction in GBM cell lines and BTIC-10 in response to RTK-Is. (**A**) U87 and HTZ-349 were cultured and treated as indicated. Whole cell protein lysates were prepared 24 hours post treatment and phosphorylation of eIF2α and expression of ATF4 were assessed by Western Blot. Actin was used as a loading control. (**B**) BTIC-10 cells were treated as indicated. Whole cell protein lysates were prepared 24 hours post treatment. Phosphorylation and expression of eIF2α was assessed by Western blot. Actin was used as loading control.

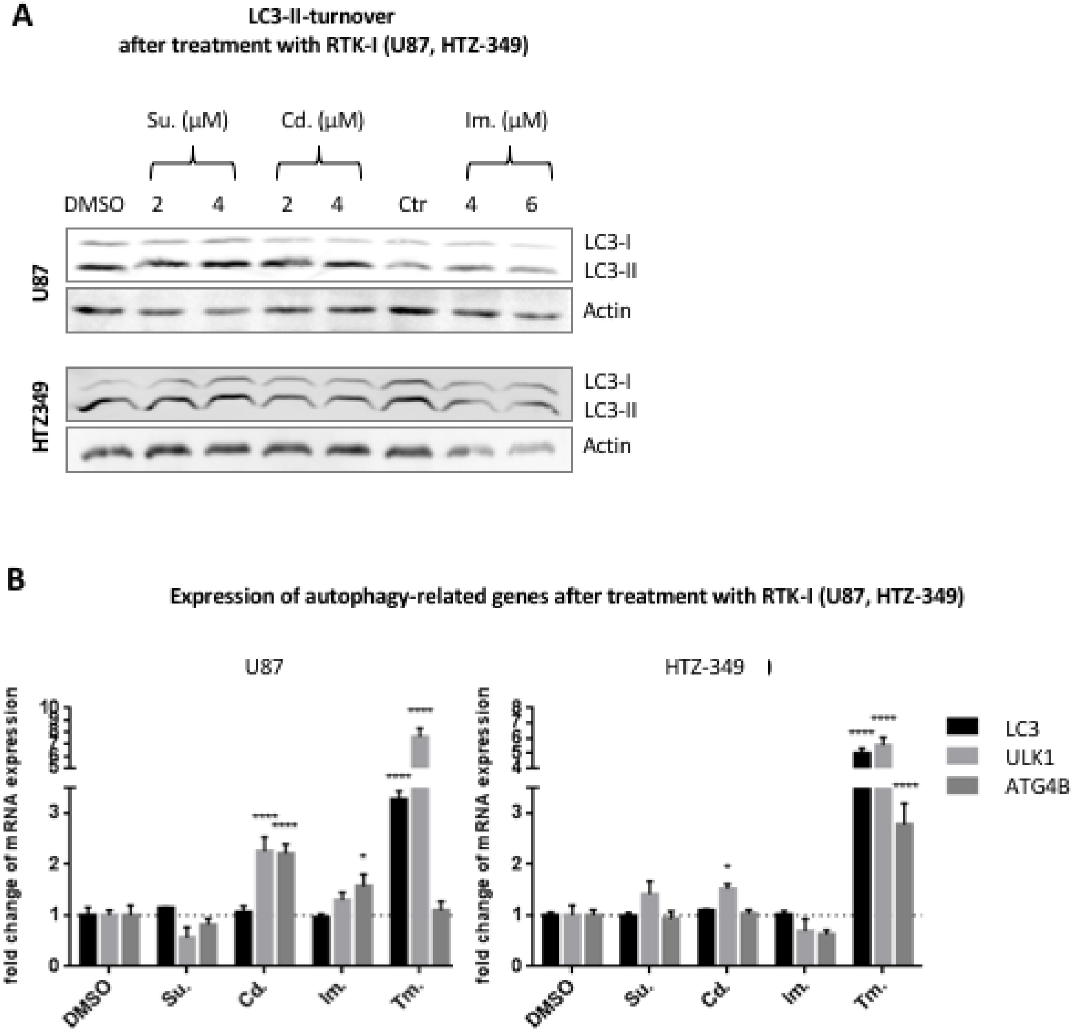

Supplementary Figure 4: Autophagic turnover and induction of autophagy-related genes after treatment with RTK-Is. (**A**) U87 and HTZ-349 were treated as indicated. Whole cell protein lysates were prepared 24 hours post treatment and LC3 expression was assessed by Western Blot. The upper LC3 signal corresponds to the LC3-I isoform and the lower signal to the LC3-II isoform. Actin was used as a loading control. (**B**) U87 and HTZ-349 were treated with 4 µM Sunitinib (Su.), 2 µM Cediranib (Ced.), 6 µM Imantinib, 0.25 µg/ml, Tunicamycin or DMSO (control) for 24 hours. Expression of autophagy-related genes was analyzed by qRT-PCR. The measurement was performed in triplicates. The ΔΔCt method was performed for relative quantification of expression levels. GAPDH was used as housekeeping gene. Data represent mean ± SD fold changes of expression relative to control treatment.

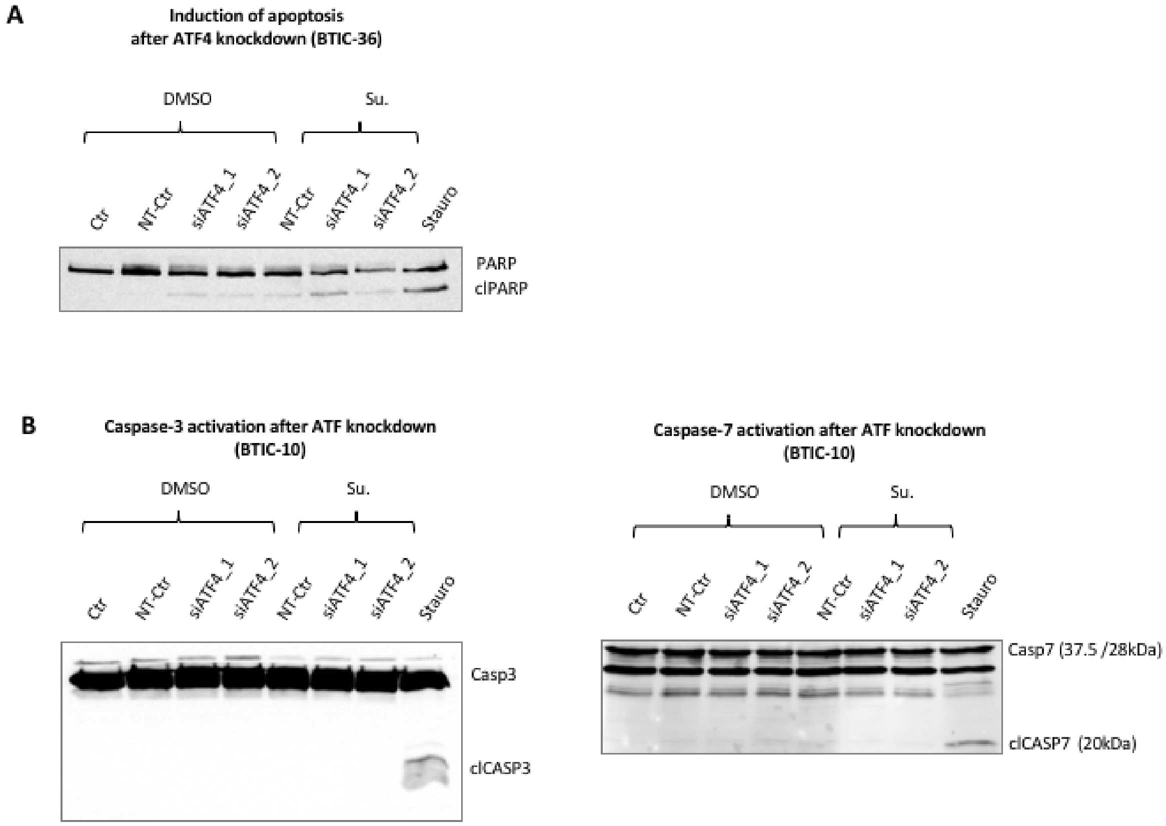

Supplementary Figure 5: PARP cleavage after ATF4 knockdown is independent of caspases. (**A**) BTIC-36 cells were transfected with siRNAs against ATF4 or non-targeting siRNA (NT-Ctr) and exposed to treatment with Sunitinib (4 µM) or DMSO (ctr) for 24 hours. Nuclear cell extracts were subjected to Western Blot analysis. Immunostaining against the PARP cleavage product (cl-PARP; 86 kDa) was performed to assess apoptosis. Staurosporine treatment (1 µM) was used as a positive control. (**B**) BTIC-10 cells were transfected with siRNAs against ATF4 or non-targeting siRNA (NT-Ctr) and exposed to treatment with Sunitinib (4 µM) or DMSO (ctr) for 24 hours. Whole cell protein lysates were subjected to Western Blot analysis. Immunostaining against full-length (35 kDa) Caspase3 and Caspase3-cleavage product (17/19 kDa) and against precursor (37.5/28kDa) Caspase7 and Caspase7 cleavage product (20 kDa) was performed to assess apoptosis. Staurosporine treatment (1 µM) was used as a positive control.
